# Draft genome sequence of *Salibacterium* sp. K-3, a halophilic Mn(II)-oxidizing bacterium

**DOI:** 10.1128/mra.01475-25

**Published:** 2026-04-23

**Authors:** Luma A. Abdulwahed, Anthony C. Greene

**Affiliations:** 1 School of Environment and Science, Griffith University 5723, Brisbane, Queensland, Australia; University of Wisconsin-Madison, Madison, Wisconsin, USA

**Keywords:** *Salibacterium* sp., Mn(II)-oxidizing bacteria, halophilic

## Abstract

*Salibacterium* sp. strain K-3 is an aerobic, halophilic Mn(II)-oxidizing bacterium isolated from hypersaline sediments of Kati Thanda–Lake Eyre, Australia. The draft genome contains 3,811,952 bp with a GC content of 47.56%, and 3,871 genes. This genome will provide insight into Mn(II) oxidation under extreme halophilic conditions.

## ANNOUNCEMENT

Microorganisms play key roles in the oxidation of Mn(II) to insoluble Mn(III/IV) oxides in aquatic and terrestrial environments (1, 2). While the mechanisms in marine and freshwater systems have been well described, comparatively little is known about Mn(II) oxidation under hypersaline conditions (3–5). Known bacterial Mn(II) oxidation pathways typically involve multicopper oxidases or heme-dependent peroxidases (6). Here, we report the draft genome sequence of *Salibacterium* sp. strain K-3 to support investigations into Mn(II) oxidation in extreme salinity.

Strain K-3 was isolated from shoreline, salt-crusted sediments of the endorheic Kati Thanda–Lake Eyre, South Australia (28°26′30.8″S, 136°58′14.2″E), collected in May 2015. Samples were stored at 4°C until processing. Enrichment cultures were established in Luria-Bertani (LB) broth (Oxoid) supplemented with 3.4 M NaCl and 1 mM MnCl₂·4H₂O. Primary isolation involved streak plating several loopfuls of a positive Mn(II)-oxidizing enrichment onto the same medium, solidified with 16 g/L bacteriological agar. A single dark-brown colony indicative of Mn(II) oxidation was selected and subcultured to purity. Mn(II) oxidation was confirmed using the leucoberbelin blue assay (7).

For genome sequencing, strain K-3 was cultivated in LB broth supplemented with 3.4 M NaCl for 48 h on an orbital shaker at 30°C. Genomic DNA was extracted using the Wizard Genomic DNA Purification Kit (Promega). Sequencing libraries were prepared using the Nextera DNA Flex Library Preparation Kit (Illumina), pooled at 2 nM per library, and sequenced on an Illumina NovaSeq 6000 platform (SP Reagent Kit v.1.5; 2 × 150 bp paired-end). Read quality was assessed using FastQC v.0.72 (8). Default parameters were used for all software unless otherwise specified. Low-quality bases (<Q20) were trimmed using Trimmomatic v.0.36.6 with a 4 bp sliding window (9). A total of 13,913,018 reads were retained and assembled *de novo* using SPAdes v.3.15.5 with Illumina-specific parameters (10). Assembly quality was assessed using QUAST v.5.3 (11), BUSCO v.5.8 (Bacillales_odb10) (12), and CheckM2 v.1.1.0 (13). Genome annotation was performed using the Prokaryotic Genome Annotation Pipeline (PGAP) v.6.10 (14) and the RAST v.2.0 Toolkit (15). Protein-coding sequences (CDSs) were predicted by Prodigal 2.6.3 (16). Taxonomic placement was assessed using the Orthologous Average Nucleotide Identity Tool (OAT) v.0.93.1 (17).

The draft genome assembly comprised 20 contigs (8 contigs >500 bp), with a total length of 3,811,952 bp and an average coverage of 380×. The GC content is 47.56%. The largest contig was 2,376,342 bp, and the N50 and L50 values were 2,376,342 bp and 1, respectively. Genome completeness indicated a high-quality assembly, with 98.2% of the 450 single-copy orthologs present and 0.52% contamination. Genome annotation identified 3,871 genes, including 3,793 CDSs, 63 tRNAs, and 11 rRNAs. There were 53 pseudogenes detected, of which 41 were attributed to incomplete coverage. ANI analysis indicated that strain K-3 shares no more than 80% identity with described genomes; the closest match was *Salibacterium halotolerans* S7 (GenBank accession FOXD00000000) at 79.6% ANI.

Strain K-3 oxidized Mn(II) under laboratory conditions. Although no clear multicopper oxidase genes were identified, the genome encodes multiple heme-dependent peroxidases and extensive oxidative stress and metal resistance systems.

## Data Availability

The draft genome sequence of *Salibacterium* sp. strain K-3 is deposited in GenBank under BioProject PRJNA1346261, BioSample SAMN52829569, and accession number JBSVZE000000000. Raw sequence reads are available in the Sequence Read Archive under accession number SRR36064364.
